# Captivity Influences the Gut Microbiome of *Rhinopithecus roxellana*

**DOI:** 10.3389/fmicb.2021.763022

**Published:** 2021-12-07

**Authors:** Xiaochen Wang, Ziming Wang, Huijuan Pan, Jiwei Qi, Dayong Li, Liye Zhang, Ying Shen, Zuofu Xiang, Ming Li

**Affiliations:** ^1^CAS Key Laboratory of Animal Ecology and Conservation Biology, Institute of Zoology, Chinese Academy of Sciences, Beijing, China; ^2^College of Life Sciences, University of Chinese Academy of Sciences, Beijing, China; ^3^School of Ecology and Nature Conservation, Beijing Forestry University, Beijing, China; ^4^Key Laboratory of Southwest China Wildlife Resources Conservation (Ministry of Education), China West Normal University, Nanchong, China; ^5^Primate Genetics Laboratory, German Primate Center, Leibniz Institute for Primate Research, Göttingen, Germany; ^6^College of Life Sciences and Technology, Central South University of Forestry and Technology, Changsha, China; ^7^Center for Excellence in Animal Evolution and Genetics, Chinese Academy of Sciences, Kunming, China

**Keywords:** *Rhinopithecus roxellana*, gut microbiome, MWAS, diet composition, captive environment

## Abstract

*Ex situ* (captivity in zoos) is regarded as an important form of conservation for endangered animals. Many studies have compared differences in the gut microbiome between captive and wild animals, but few have explained those differences at the functional level due to the limited amount of 16S rRNA data. Here, we compared the gut microbiome of captive and wild *Rhinopithecus roxellana*, whose high degree of dietary specificity makes it a good subject to observe the effects of the captive environment on their gut microbiome, by performing a metagenome-wide association study (MWAS). The Chao1 index was significantly higher in the captive *R. roxellana* cohort than in the wild cohort, and the Shannon index of captive *R. roxellana* was higher than that of the wild cohort but the difference was not significant. A significantly increased ratio of *Prevotella*/*Bacteroides*, which revealed an increased ability to digest simple carbohydrates, was found in the captive cohort. A significant decrease in the abundance of Firmicutes and enrichment of genes related to the pentose phosphate pathway were noted in the captive cohort, indicating a decreased ability of captive monkeys to digest fiber. Additionally, genes required for glutamate biosynthesis were also significantly more abundant in the captive cohort than in the wild cohort. These changes in the gut microbiome correspond to changes in the composition of the diet in captive animals, which has more simple carbohydrates and less crude fiber and protein than the diet of the wild animals. In addition, more unique bacteria in captive *R. roxellana* were involved in antibiotic resistance (*Acinetobacter*) and diarrhea (*Desulfovibrio piger*), and in the prevention of diarrhea (*Phascolarctobacterium succinatutens*) caused by *Clostridioides difficile*. Accordingly, our data reveal the cause-and-effect relationships between changes in the exact dietary composition and changes in the gut microbiome on both the structural and functional levels by comparing of captive and wild *R. roxellana*.

## Introduction

The golden snub-nosed monkey (*Rhinopithecus roxellana*) is an endangered colobine species endemic to China (Kirkpatrick and Grueter, 2010), and this species is highly folivorous and exploits a diet composed principally of leaves, seeds, bark, and lichen (Zhou et al., 2014). At present, *R. roxellana* habitats in some isolated mountains, such as the Qionglai, Minshan, Qinling, and Daba Mountains of Central China, have more than 25,000 individuals (Luo et al., 2012; Zhou et al., 2016). As an iconic endangered species and flagship species for tourism in China due to its golden coat, blue facial coloration, snub nose and specialized life history, the Chinese government launched many conservation strategies, such as *in situ* (natural reserves) and *ex situ* strategies (captivity in zoos), to protect this species. Currently, more than 400 individuals of *R. roxellana* are being raised in captivity (Xiang et al., 2017).

Substantial differences between diets of colobine primates in captivity and in the wild have been identified. Diets of captive colobine primates contain lower amounts of crude fiber (11–35%) than natural diets (up to 52%) (Nijboer and Clauss, 2006). Further research has shown that captive *R. roxellana* have a lower intake of crude fiber (15%) and protein (13%) and a higher intake of non-structural carbohydrates (60%) and fat (12%) than wild monkeys (Chen et al., 2018; Guo et al., 2018). Some captive *R. roxellana* (Louguantai) cohorts exhibit much lower protein intake (mean: 9.2%) than wild cohorts (Chen et al., 2018). Additionally, captive monkeys have more chances of being exposed to humans, resulting in infection with viruses and bacteria from humans. These changes are thought to be associated with changes in the gut microbiome of captive monkeys, which in turn are related to various diseases, such as gastrointestinal (GI) problems (Amato et al., 2016; Zhu et al., 2018; Hale et al., 2019). As a leaf-eating non-human primate, *R. roxellana* has a specialized diet and habitat (Luo et al., 2012; Zhou et al., 2014, 2016), and thus it is a good subject to assess the link between their gut microbiome and the changing environment.

The gut microbiome, the trillions of bacteria which exists in the GI tract, plays an important roles in host metabolism and immunity (Clayton et al., 2018; West et al., 2019). Multiple studies have indicated that the diet and surrounding environment exert obvious effects on the gut microbiome (Clayton et al., 2018; Baj et al., 2019; West et al., 2019) because the gut microbiome is highly flexible, enabling the host to respond rapidly to changes in the environment (David et al., 2014; Suzuki and Ley, 2020). Captivity or lifestyle disruption causes primates to lose native microbiome (Frankel et al., 2019) and converge along an axis toward the modern human microbiome (Clayton et al., 2016; Campbell et al., 2020). Previous studies have reported significant differences in the gut microbiome between captive and wild *Rhinopithecus brelichi* (Hale et al., 2019). Therefore, we postulated that significant differences would exist in the gut microbiome between captive and wild *R. roxellana* and that these differences were probably related to the changing diet and environment. However, little is known about these differences at both the taxonomic and functional levels of the gut microbiome between captive and wild *R. roxellana* (Su et al., 2016; Hale et al., 2018; Zhu et al., 2018). Even fewer studies based on metagenomic data have elucidated the characteristics and function of the gut microbiome at the species and gene levels.

Captivity and loss of dietary fiber in non-human primates are associated with loss of native gut microbiome and convergence toward the modern human microbiota (Clayton et al., 2016). Therefore, we sequenced the metagenomic data from 28 individuals, including captive *R. roxellana*, wild *R. roxellana*, and captive *Macaca mulatta*, using whole-genome shotgun sequencing, and downloaded the metagenomic data for humans from the National Center for Biotechnology Information (NCBI) database (Qin et al., 2012). We compared the differences between captive and wild *R. roxellana* cohorts at both taxonomic and functional levels by performing a metagenome-wide association study (MWAS) (Kishikawa et al., 2020) to investigate the loss of native gut microbiome of captive cohorts. We further look for the similar traits in microbiome of captive *R. roxellana*, *captive M. mulatta* and human cohorts to explore whether convergence toward the modern human microbiome occurred in the gut microbiome of captive primates.

## Materials and Methods

### Study Subjects and Samples Collection

We collected fecal samples from 9 *R. roxellana* individuals (CRr) from Shanghai Wild Animal Park and 10 *M. mulatta* individuals (CMm) from Beijing Wildlife Park (Supplementary Table 1A) as our two captive cohorts. We further collected fecal samples from 9 *R. roxellana* individuals (WRr) from Baihe National Nature Reserve, Sichuan Province (Supplementary Table 1A) as the wild cohort. Because *R. roxellana* is endangered and has a small population, samples are difficult to obtain. Therefore, we used shotgun metagenomes with more information than 16S rRNA data to explore the gut microbiome. Each cohort in our study contained at least 9 samples which can generally represents a group well (Quince et al., 2017; Knight et al., 2018). All fresh feces were collected in 3 ml of RNAlater immediately after defecation (Vlckova et al., 2012; Blekhman et al., 2016). These samples were transported on ice within 1 week and then stored at −80°C until DNA extraction at the Institute of Zoology, Chinese Academy of Sciences (CAS). For the human cohort (Hum), we downloaded fecal microbiome information, which includes both genus info and K-numbers info, of nine healthy Chinese individuals as controls from published articles (Qin et al., 2012) (Supplementary Table 1A). We added human (Hum) and captive *M. mulatta* (CMm) samples as supplementary cohorts to verify the effects of the captive environment on the gut microbiome. In our study, we postulated that captive environmental factors include diet changes, human contact, the use of antibiotics, and other factors.

### DNA Extraction and Sequencing

Microbial DNA was extracted from the fecal samples using the QIAamp DNA stool mini kit (Qiagen, Valencia, CA, United States) according to the standard protocol. The quality and quantity of the DNA were determined with a Nanodrop (ND-1000) spectrophotometer (Nanodrop Technologies, Wilmington, DE, United States) and agarose gel electrophoresis. DNA samples were stored at −20°C untill use. Shotgun sequencing was performed using an Illumina NovaSeq 6000, with at least 10 Gb per sample. We filtered the raw data using Trimmomatic v0.36 (Bolger et al., 2014) to trim low-quality reads: 3′ tailing sequences were removed when the average quality over a 4-b sliding window was less than 20, and reads less than 70 bp were discarded. Then, we used the genomes of *R. roxellana* (assembly ASM756505v1) and *Homo sapiens* (assembly GRCh38.p13) to remove contamination and obtain clean data with bowtie2 v2.3.5 (Langmead and Salzberg, 2012). After the removal of low-quality and contaminating reads, an average of 11.6 Gb of high-quality non-host sequences were obtained from each sample in the CRr, WRr, and CMm cohorts (Supplementary Table 1B).

### Determination of the Relative Abundance of Taxonomic and Functional Terms

Taxonomic profiles at the species level were generated using the (MG)-based operational taxonomic units (mOTUs) profiler (v2.0.0) (Milanese et al., 2019) with the following parameters: −l 75; −g 2; and −c. mOTUs profiles were first converted to relative abundance to account for the library size. Afterward, the relative abundance at the genus, family, order, class, and phylum levels was determined by mOTUs using the parameter −k. Then, taxonomic terms that did not exceed a maximum relative abundance of 1 × 10^–4^ were excluded from further analysis, together with taxonomic terms accounting for less than 20% of the samples in each cohort (Wirbel et al., 2019). After selection, we assessed 396 taxonomic terms (13 phyla, 20 classes, 27 orders, 37 families, 61 genera, and 238 species) for CRr and WRr.

We applied single-sample metagenomic assembly and functional annotation to compare the gut microbiome at the gene level and functional level between the captive-wild cohorts. Briefly, assemblies were produced with MEGAHIT software (v1.2.6) (Li et al., 2015), and gene identification was performed on contigs longer than 300 bp using MetaGeneMark (Zhu et al., 2010). Next, we annotated the contigs with Kyoto Encyclopedia of Genes and Genomes (KEGG, v50) (Kanehisa et al., 2016) *via* DIAMOND (v0.9.24) with the parameters −d −q −e 1e−5 −k 1 (Buchfink et al., 2015). We further calculated the relative abundance of K-numbers (level 3 pathways) and removed the K-numbers detected (i) in less than 20% of the samples from each cohort or (ii) in no sample from either cohort. Specifically, K-numbers that did not exceed a maximum relative abundance of 1 × 10^–6^ were excluded from further analysis (Wirbel et al., 2019). After gene selection, we assessed 261,182 genes annotated by the KEGG gene database and 4,895 K-numbers (Supplementary Table 3) for CRr and WRr.

### Correlation Test, Alpha Diversity, and Beta Diversity Analyses and Hierarchical Tree

We calculated the correlations among the four cohorts using the R “vegan” package based on ANOSIM. Then, we used the abundance/relative abundance at the genus level to calculate the alpha diversity, beta diversity and a hierarchical tree. Two estimators of the alpha diversity index, Chao1, and Shannon indices, for the four cohorts were calculated using the R package “vegan” (Oksanen et al., 2019). The Shannon diversity index accounts for the richness and evenness of species distribution, whereas the Chao1 index extrapolates the number of rare taxa that may have been accounted for with deeper sampling. We further compared each estimator by performing the Wilcoxon rank sum test with the R “coin” package (Hothorn et al., 2006), and the *p*-values were adjusted using the Benjamini–Hochberg false discovery rate (FDR) method (Benjamini and Hochberg, 1995). We performed principal component analysis (PCoA) based on Bray–Curtis dissimilarities using the R “vegan” package for all cohorts (Oksanen et al., 2019), which was visualized with the “ggplot2” package.

We constructed a hierarchical tree of the gut microbiome for the four cohorts and a phylogenetic tree for the three hosts to compare the effect of environmental and phylogenetic factors on the gut microbiome. We used the R “vegan” package (Oksanen et al., 2019) to calculate divergences among the four cohorts based on Bray–Curtis dissimilarity to build the hierarchical tree. The phylogenetic tree was constructed based on the time tree available at http://timetree.org/ (Kumar et al., 2017).

### Captive–Wild Association Tests

Captive–wild association tests at both the taxonomic and functional levels were performed using the generalized linear model (GLM) in the R package “glm2” (v1.2.1) (Kishikawa et al., 2020), and *p*-values were adjusted using the Benjamini–Hochberg FDR method (Gao et al., 2019). Twenty-five taxonomic terms that were significantly different [*p(FDR)* < 0.005] between wild-captive *R. roxellana* were visualized in a heatmap using the R “pheatmap” package (Kolde, 2018) (Supplementary Table 4 and Figure 2D). Forty K-numbers that were significantly different between wild and captive *R. roxellana* [*p(FDR)* < 1e-5] were visualized in a volcano plot using the R “ggrepel” package (Supplementary Table 3 and Figure 3A). In the volcano plot, the *x*-axis indicates the beta value of the GLM as the effect size. The *y*-axis indicates the observed −log10 (*FDR-corrected p-values*). We further visualized the KEGG pathways enriched in the 40 K-numbers in a stem diagram using the R “graphlan” package (v1.1.3) (Asnicar et al., 2015). Ten pathways were identified in the enrichment analysis based on the KEGG database. In addition, all pairwise comparisons in this study were calculated using the Wilcoxon rank sum test with a FDR correction for multiple testing correction, except for the GLM analysis.

**FIGURE 1:**
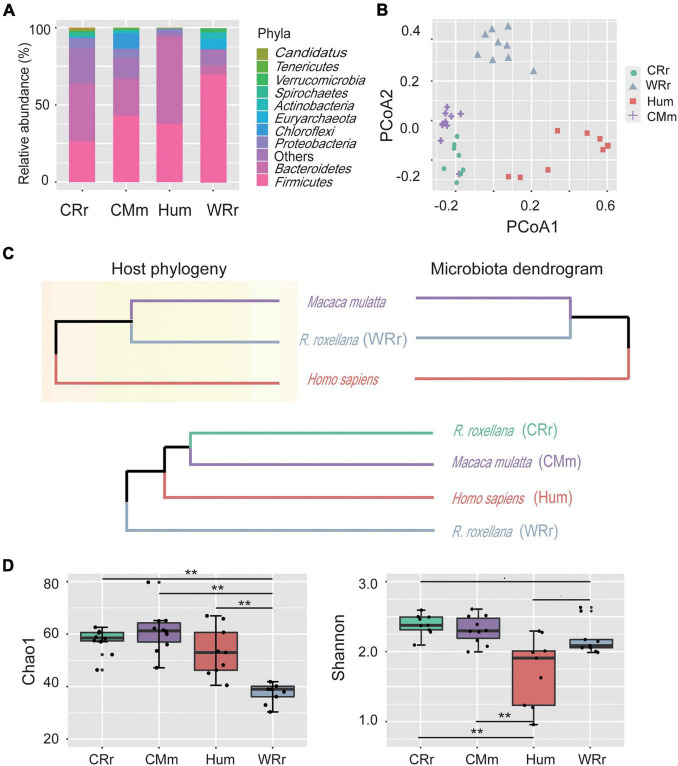
Community constituents, structure, richness, and diversity of the gut microbiome among all cohorts. **(A)** Compositional bar plot of the ten most abundant phyla in each cohort (WRr: wild *R. roxellana*; CRr: captive *R. roxellana*; Hum: humans; CMm: captive *M. mulatta*). **(B)** PCoA plot of the gut microbiome community composition in the four cohorts at the genus level. **(C)** Comparison of the host phylogeny (upper left panel; assembled using http://timetree.org/) and their hierarchical tree (upper right panel). The gut microbiome dendrogram of the four cohorts (lower panel). **(D)** Alpha diversity of the gut microbiome in the four cohorts and [*p*(FDR)*-value*] between cohorts. Two asterisk indicates significant differences (*p*(FDR)-value < 0.01). Panel **(A–D)** indicate the tremendous effects of captivity and lifestyle on captive monkeys, and the gut microbiome of captive monkeys was more similar to that of humans than to that of wild monkeys.

**FIGURE 2:**
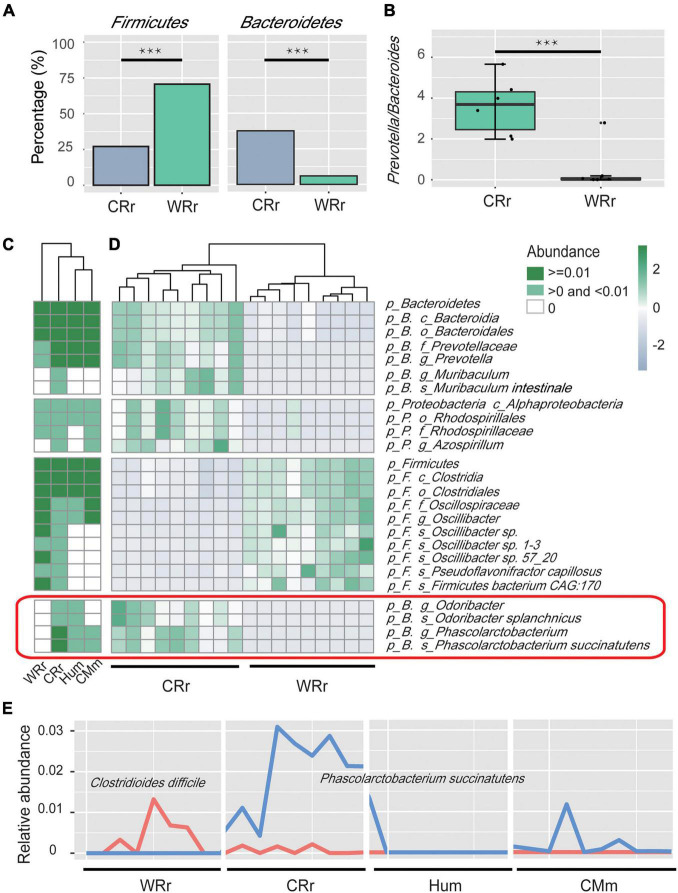
Microbial community profiles of captive and wild *R. roxellana* microbiome. **(A)** Bar plots showing the relative abundance of Firmicutes and Bacteroidetes between WRr and CRr samples. Three asterisk indicates significant differences (*p*(FDR)-value < 0.001). **(B)** Box plots showing the *Prevotella*/*Bacteroides* (P/B) ratio of WRr and CRr samples. **(C)** The average relative abundance of taxa in the four cohorts (white = average relative abundance = 0; light green = average relative abundance < 0.01; dark green = average relative abundance > 0.01). **(D)** A heatmap showing the taxonomic terms that were significantly different [*p(FDR)* < 0.005] between wild and captive *R. roxellana*. Bacteria in the red box were shared by CRr, Hum, and CMm samples but not WRr samples. **(E)** The distribution of the relative abundances of *C. difficile* and *P. succinatutens* among the four cohorts. Panel **(A–E)** indicate the significant differences in bacterial communities between CRr and WRr samples, and the captive populations share some fecal microbes with humans.

**FIGURE 3:**
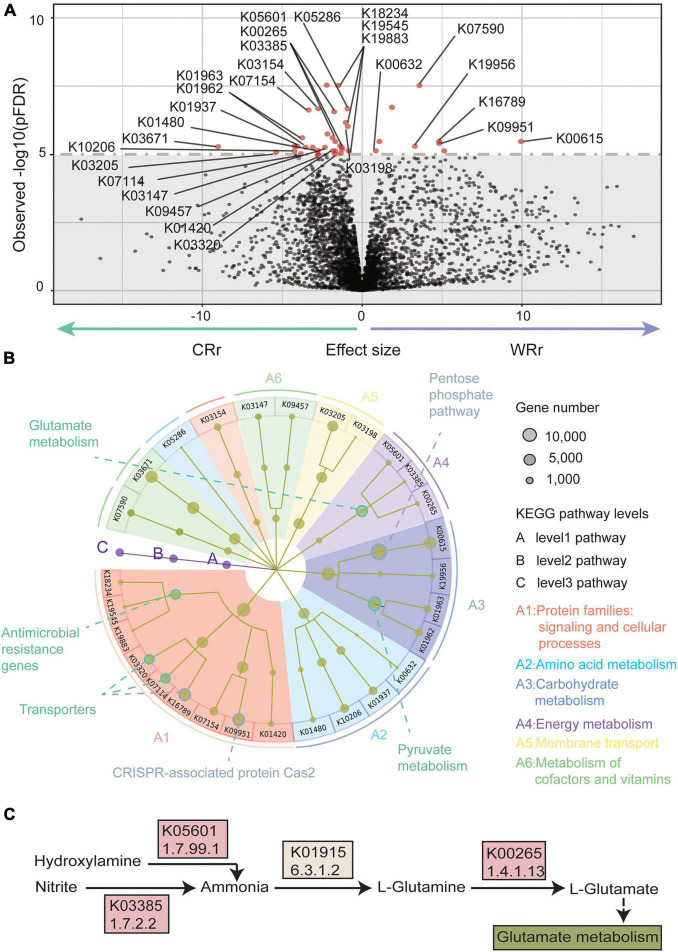
Metagenome-wide association study results of the wild-captive *R. roxellana* gene association test. **(A)** A volcano plot of the K-numbers based on the KEGG database. In the volcano plot, the *x*-axis indicates beta value of the GLM as the effect size. The *y*-axis indicates observed –log10 [*p(FDR)*-values]. The horizontal dotted line indicates *p(FDR)* = 1e-5. There were 40 K-numbers with *p(FDR)* < 1e-5 are plotted as red dots, and other clades are plotted as black dots. **(B)** System diagram of KEGG pathways enriched with the 40 K-numbers highlighted in **(A)**. The three levels are defined as A, B, and C and described from the inner layer out. The size of the dots represents the number of genes. The eight pathways with significant enrichment are outlined by circles (green: pathways with significantly different abundance in CRr samples; gray: pathways with significantly different abundance in WRr samples). **(C)** A pathway diagram showing the K-numbers associated with glutamate metabolism. Panel **(A–C)** show the significantly differentially abundant pathways between captive and wild *R. roxellana* and the abundance K-numbers associated with L-glutamate in the captive population.

## Results

### Captivity Changes the Microbiome Constituents and Community Structure of *Rhinopithecus roxellana*

We performed whole-genome shotgun sequencing of a total of 18 *R. roxellana* fecal samples (9 CRr and 9 WRr), 10 captive *M. mulatta* (CMm) samples and 9 samples from healthy Chinese individuals (Hum), for which the taxonomy and KEGG annotation information was downloaded from NCBI (Qin et al., 2012) (Supplementary Table 1A). The average size of the whole-genome shotgun sequencing data from the three monkey cohorts was greater than 11.16 Gb, and all samples passed stringent quality control (Supplementary Table 1B). Thirteen phyla, 20 classes, 27 orders, 37 families, 61 genera, and 238 species were identified by MOTUs2 from 18 metagenomes of *R. roxellana* (Supplementary Table 2). The two most abundant phyla were Firmicutes (WRr: 70.7 ± 0.11%; CRr: 27.1 ± 0.1%) and Bacteroidetes (WRr: 5.9 ± 0.07%; CRr: 37.6 ± 0.09%) in both captive and wild *R. roxellana* (Supplementary Figure 1 and Figure 1A).

Correlation analysis (Supplementary Figure 2) and principal coordinate analysis (PCoA; Figure 1B) showed distinct differences in diversity among the gut microbiome of the four cohorts. The PCoA results indicated that the human cohort had the most variability, whereas the captive cohorts showed the lowest variability. Captive *R. roxellana* and captive *M. mulatta* clustered more closely and had lower Bray–Curtis distances with the human cohorts than wild *R. roxellana*. The host phylogeny and hierarchical trees of the 3 hosts and their gut microbiome composition were constructed to compare the effects of environmental and phylogenetic factors on the gut microbiome (Supplementary Figure 3 and Figure 1C). The phylogenetic tree and the hierarchical trees presented a mirror image (Figure 1C, upper panel), confirming a correlation between the phylogeny and the gut microbiome. Similar to the PCoA results, the captive *R. roxellana* were clustered with captive *M. mulatta* and humans rather than with the wild *R. roxellana* (Supplementary Figure 3 and Figure 1C, lower panel). Similar results were reported among captive chimpanzees and gorillas compared with their wild cohorts (Campbell et al., 2020). Based on the comparison of the gut microbiome in the wild *R. roxellana* cohort, all these results showed that the captive environment tremendously altered the composition of the gut microbiome.

We further calculated significant differences in community richness based on the abundance at the genus level across all cohorts (Figure 1D), as estimated by Chao1 and Shannon index. Microbial diversity based on both Chao1 (accounts for rare species) and Shannon (accounts for species richness and evenness) indices was significantly increased in captive *R. roxellana* compared with the wild cohort [Figure 1D; Chao1: *p(FDR)* < 0.005; Shannon: *p(FDR)* < 0.1]. In addition, the Shannon index, but not Chao1 index, of captive *R. roxellana* was significantly increased compared with human cohorts [Figure 1D; *p(FDR)* < 0.01]. No significant differences in the Chao1 and Shannon diversity indices were observed between captive *R. roxellana* and captive *M. mulatta*. Thus, more rare species were present in the gut microbiome of captive monkeys and human than in the wild cohort.

### An Increased Ratio of *Prevotella*/*Bacteroides* in Captive *Rhinopithecus roxellana*

We used a GLM (Kishikawa et al., 2020) to identify the key bacteria responsible for the differences in the relative abundances at each taxonomy level between captive and wild *R. roxellana*. The results indicated differences in the abundance of 48 taxa between captive and wild *R. roxellana* [Supplementary Table 4A; overall *p(FDR)* < 0.005]. At the phylum level, an increase in Bacteroidetes and a decrease in Firmicutes abundance in the gut microbiome were observed in captive *R. roxellana* [Figures 2A,D; both *p(FDR)* < 0.0001]. Bacteroidetes are considered primary degraders of polysaccharides (Lapebie et al., 2019), and Firmicutes are known to utilize xylose (Gu et al., 2010), such as the crude fiber present in the diet of *R. roxellana*.

At the genus level, the *Prevotella*/*Bacteroides* (P/B) ratio was also significantly increased in captive *R. roxellana* (Figure 2B; Wilcoxon rank sum test *p* < 0.001). An increasing P/B ratio has been reported to be related to the loss of fiber digestion capability (Chen et al., 2017). *Prevotella* degrade simple sugars and carbohydrates, which are two staples of the captive monkey diet (Hale et al., 2019). Based on these results, the gut microbiome of captive *R. roxellana* lost the ability to digest fiber and increased their ability to digest simple carbohydrates.

For bacteria (5 genera and 3 species) that were only present in captive *R. roxellana* compared to the wild cohort, heatmaps of the 4 cohorts (WRr, CRr, Hum, and CMm) were established to show the effect of the human environment on gut microbiome (Supplementary Figure 4 and Figures 2C,D). Among those bacteria, *Phascolarctobacterium* (*P. succinatutens*), *Acinetobacter*, and *Desulfovibrio* (*D. piger*) were identified in the CRr, Hum, and CMm cohorts but not in the WRr cohort. *P. succinatutens* was reported to effectively inhibit the colonization of *Clostridioides difficile* (Nagao-Kitamoto et al., 2020), which can cause severe, potentially life-threatening intestinal inflammation (Britton and Young, 2014). Therefore, we compared the abundance of *C. difficile* between WRr and CRr (Figure 2E). *C. difficile* was more abundant in WRr [*p(FDR)* = 0.24] than in CRr, indicating that captive *R. roxellana* may be less likely to have diarrhea caused by *C. difficile* than the wild cohort. In addition, *Acinetobacter*, which is known for its broad-spectrum antibiotic resistance (Towner, 2009; Fishbain and Peleg, 2010), and *D. piger*, which is related to inflammatory bowel disease (IBD) (Loubinoux et al., 2002), were more abundant in CRr than WRr, showing that captive *R. roxellana* may have higher resistance and a higher risk of diarrhea caused by *D. piger.* These bacteria, which were not detected in the WRr cohort but were observed in the CRr, Hum, and CMm cohorts, were probably transplanted into the gut microbiome of captive *R. roxellana* through the captive environment.

### Captive *Rhinopithecus roxellana* Exhibited Increased Abundance of Genes Involved in Glutamate Metabolism

We annotated 261,182 genes and 4,895 K-numbers (Supplementary Table 3) from both captive and wild *R. roxellana* using the KEGG database (Kanehisa and Goto, 2000). Forty K-numbers (level 3 pathways) [Figure 3A; overall *p(FDR)* < 0.00001] were significantly different between the wild and captive *R. roxellana* cohorts, according to the GLM analysis (Kishikawa et al., 2020). Twenty-eight of the 40 K-numbers were enriched in 18 KEGG orthology (KO) (level 2 pathways) terms and 10 pathways (level 1 pathways) (Figure 3B). Of the level 1 pathways, the top 4 enriched pathways were signaling and cellular processes (9 K-numbers), amino acid metabolism (4 K-numbers), carbohydrate metabolism (4 K-numbers), and energy metabolism (3 K-numbers).

In the signaling and cellular processes pathway, genes related to antimicrobial resistance, which is associated with resistance to a variety of antibiotics, were significantly more abundant in the captive *R. roxellana* cohort than in the wild cohort (K19883, 2.3.1.82/2.7.1.190; K19545; K18234, 2.3.1.-), indicating that captive animals have higher resistance levels. In the amino acid metabolism pathway, genes involved in lysine biosynthesis (K10206) and arginine and proline metabolism (K01480) were significantly more abundant in captive *R. roxellana*. In the carbohydrate metabolism pathway, pyruvate metabolism related genes, which are involved in fatty acid biosynthesis (Schujman et al., 2008), were also more abundant in captive *R. roxellana* (K01962/K01963, 2.1.3.15/6.4.1.2). However, the genes involved in the pentose phosphate pathway, which have previously been implicated in fiber metabolism (Thurston et al., 1994; Vatanen et al., 2018), were significantly less abundant in captive *R. roxellana* (K00615, EC:2.2.1.1).

In the energy metabolism pathway, genes related to glutamate (Glu) metabolism were significantly more abundant in the gut microbiome of captive *R. roxellana* than in wild *R. roxellana* (K05601, 1.7.99.1; K00265, 1.4.1.13; K03385, 1.7.2.2). The main function of these genes is to synthesize Glu (Figure 3C). Our results suggest that the gut microbiome of captive monkeys has the potential to synthesize more Glu than that of wild monkeys. Glu is among the most abundant amino acids (8–10%) found in dietary proteins (Rangan, 2008). In the gut, Glu is derived from dietary proteins, free Glu in food additives and bacterial synthesis (Mazzoli and Pessione, 2016; Tome, 2018). Additionally, Glu is an important fuel for intestinal tissue, is involved in gut protein metabolism, and is the precursor of different important molecules produced within the intestinal mucosa (Mazzoli and Pessione, 2016; Tome, 2018).

## Discussion

This study is the first to reveal the characteristics of the gut microbiome of *R. roxellana* by analyzing metagenome data. Our findings indicated that changes in the *R. roxellana* gut microbiome may help animals cope with changes in the composition of the diet in captivity and different habitats in captive environments. First, we found that captivity significantly altered the components and community structure of the gut microbiome in *R. roxellana*. Second, an increased P/B ratio and abundance of genes involved in Glu metabolism in captivity suggested that the structure and function of the gut microbiome in *R. roxellana* were altered according to the diet. Finally, we further identified bacteria that might be related to diarrhea, but specific strains may help prevent diarrhea.

Both the results of beta and alpha diversity analyses showed noticeable effects of captive environment on the gut microbiome of *R. roxellana*. In our study, captive *R. roxellana* clustered more closely to captive *M. mulatta* and humans than wild *R. roxellana* (Figures 1B,C). Similar results were reported in other non-human primates (NHPs) (Clayton et al., 2016; Frankel et al., 2019; Campbell et al., 2020). Captivity or lifestyle disruption causes primates to lose native microbiome (Frankel et al., 2019) and converge along an axis toward the modern human microbiota (Clayton et al., 2016; Campbell et al., 2020). We further found a greater abundance of rare bacterial species and higher richness and diversity in the captive *R. roxellana* cohort than in the wild cohort (Figure 1D). Similar results have been reported that captive chimpanzee and captive gorillas tend to have higher richness than their wild cohorts (Campbell et al., 2020). This differences may be due to changes in the diet, increased human exposure, and increased use of antibiotics in captive cohorts compared to natural cohorts (Grassotti et al., 2018; Tsukayama et al., 2018).

Some studies have confirmed that the diet of animals in captivity contains more simple carbohydrates and less crude fiber and protein than the diet of the wild cohort (Nijboer and Clauss, 2006; Chen et al., 2018; Guo et al., 2018; Liu et al., 2018; Zhu et al., 2018). Studies have showed that the gut microbiome responds to altered diets (Muegge et al., 2011; David et al., 2014; Baniel et al., 2021). We observed a decreased abundance of Firmicutes (Figure 2A) and genes involved in the pentose phosphate pathway (K00615) (Figures 3A,B) in captive *R. roxellana* compared with the wild cohort. Firmicutes are known to utilize fiber and cellulose (Gu et al., 2010), and genes involved in the pentose phosphate pathway have been implicated previously in fiber metabolism (Thurston et al., 1994; Vatanen et al., 2018). A lower abundance of Firmicutes was also detected in captive herbivorous mammals, such as *R. brelichi*, *Pseudois nayaur*, and *Moschus chrysogaster* (Chi et al., 2019; Hale et al., 2019; Sun et al., 2019). A significant decrease in the abundance of Firmicutes was found in humans who do not consume sufficient crude fiber (Nobel et al., 2018; Tanes et al., 2021). We propose that the reduction in the abundance of Firmicutes and genes involved in the breakdown of crude fiber may be because the diet of captive animals contains less crude fiber than the natural diet.

Bacteroidetes, which promotes the digestion and decomposition of polysaccharides and proteins (Spence et al., 2006), is the most abundant phylum in captive *R. roxellana*. The efficiency of simple carbohydrate digestion in animals raised in captivity seems to rely on *Prevotella* (Bacteroidetes phylum). *Prevotella* mainly digests non-cellulosic polysaccharides and pectin (Flint et al., 2012; White et al., 2014). Similar results have been reported in healthy captive *R. roxellana* (Zhu et al., 2018), but a study showed that Bacteroidetes was not the most abundant phylum in captive *R. brelichi*, but the second most abundant phylum after Firmicutes (Hale et al., 2019). Our study also found that the *Prevotella* abundance and P/B ratio increased in captive *R. roxellana* (Figures 2B,D), indicating an increased capability of digesting simple carbohydrates and a decreased capability of digesting crude fiber, respectively (Chen et al., 2017). Similar results have been reported in *R. brelichi*, chimpanzee and gorillas, in which the captive cohort exhibited a greater abundance of *Prevotella* than the wild cohort (Hale et al., 2019). The explanation for this phenomenon may be the inadequate intake of crude fiber and excess intake of simple carbohydrates by captive monkeys.

We suggested that these changes in the gut microbiome of captive *R. roxellana* may be related to excess consumption of simple carbohydrates and insufficient crude fiber intake. Studies have shown that high-fiber and low-sugar diets in the captive environment promote naturalized foraging and activity patterns of captive animals in great apes (Cabana et al., 2017) and lemurs (Schwitzer, 2008; Greene et al., 2020). Furthermore, boosting fiber intake and/or limiting sugar intake improves the health of captive animals by variably reducing obesity, diabetes, cataracts, fatty liver, parasite burden, and serum insulin and cholesterol, while improving body and coat conditions in Javan slow loris (Cabana et al., 2019). Considering that *R. roxellana* is a leaf-eating animal, we postulate that increasing the crude fiber content and limiting simple carbohydrates in the diet may improve the health of captive animals.

The significant difference in the abundance of amino acid synthetase might be related to the decreased protein intake of captive monkeys. A study reported higher amino acid synthetase gene in the gut microbiome of herbivores compared to carnivores (Muegge et al., 2011), and Glu metabolism is particularly illustrative of these trends. Similar results were found in our study, in which amino acid synthetases (Glu and lysine) were significantly more abundant in the captive *R. roxellana* cohort than in the wild cohort (Figures 3A,B). In particular, genes involved in Glu biosynthesis (K05601/K03385/K00265) were significantly more abundant in captive *R. roxellana* compared to the wild cohort, indicating the enhanced ability of the gut microbiome of captive cohorts to synthesize amino acids. Another study further reported that genes involved in Glu biosynthesis were significantly enriched in the gut microbiome of humans with a plant-based diet compared with humans with an animal-based diet, which might be related to the marked difference in protein intake between those two cohorts (David et al., 2014). Considering the lack of protein intake by the captive animals, we propose that the diet of *R. roxellana* raised in captivity should be supplemented with plants with a higher protein content to promote their nutritional balance.

Furthermore, we found that unique bacteria (*Acinetobacter* and *D. piger*) related to antibiotic resistance and IBD (Loubinoux et al., 2002; Towner, 2009; Fishbain and Peleg, 2010; Song et al., 2017) were more abundant in CRr, CMu, and Hum but not in WRr (Supplementary Figure 4). Studies have detected these bacteria in the stomach microbiome of captive *R. roxellana* (Zhou et al., 2014), and an higher abundance was observed in captive *R. roxellana* experiencing diarrhea than in healthy captive *R. roxellana* (Jian et al., 2015; Zhu et al., 2018). In this study, we found that these bacteria were also abundant in the human and *M. mulatta* cohorts but not in the wild *R. roxellana* cohort (Supplementary Figure 4), indicating that these bacteria might transmit to captive monkeys through their environment. It had been reported that captivity and lifestyle changes associated with human contact potentially lead to marked changes in the resistome of primate gut communities (Grassotti et al., 2018; Tsukayama et al., 2018). These results also suggested that captive *R. roxellana* may have a higher risk of diarrhea, which is associated with *D. piger*, than the wild cohort.

Although captivity seems to make captive *R. roxellana* to have more unique bacteria that could be harmful to the host, we found there were some bacteria that might be beneficial to the host. We found bacteria exist in the gut microbiome of captive *R. roxellana* which might help the host suppress diarrhea which caused by *C. difficile*. Studies have elucidated that *C. difficile* causes severe, potentially life-threatening intestinal inflammation (Britton and Young, 2014), but *P. succinatutens* prevents *C. difficile* growth (Nagao-Kitamoto et al., 2020). The abundance of *P. succinatutens* has been found to be lower in individuals with diarrhea than in healthy individuals (Morgan et al., 2012; Jian et al., 2015). However, in this study, we observed a higher abundance of *P. succinatutens* and a lower abundance of *C. difficile* [*p(FDR)* = 0.24] in captive *R. roxellana* than in wild *R. roxellana* (Figures 2C–E), which was also abundant in humans and captive *Macaca*, showing the positive effects of captivity on the gut microbiome of captive NHPs.

## Conclusion

In summary, the gut microbiome of *R. roxellana* was more substantially affected by the captive environment than phylogenetic factors, especially diet changes. We compared the gut microbiome of captive and wild cohorts at both the taxonomic and functional levels. We found that the gut microbiome of captive *R. roxellana* tended to have a weaker ability to digest crude fiber, a strengthened ability to digest simple carbohydrates and an increased ability to synthesize amino acids. This phenomenon might be due to the changes in the dietary composition in the captive environment, which contains more simple carbohydrates and less crude fiber and protein than the natural diet. In addition, we found that captive *R. roxellana* had more unique bacteria in their gut microbiome, which were associated with not only antibiotic resistance (*Acinetobacter*) and diarrhea (*D. piger*), but also the prevention of certain types of diarrhea (*P. succinatutens*). In our study, we observed the positive role of gut microbiome in host diet adaptation in captivity, as well as the substantial negative and positive effects of captivity on the gut microbiome, showing the complex interactions between gut microbiome and the environment.

## Data Availability Statement

The datasets presented in this study can be found in online repositories. The names of the repository/repositories and accession number(s) can be found below: NCBI, PRJNA718160.

## Ethics Statement

The animal study was reviewed and approved by the Committee for Animal Experiments of the Institute of Zoology, Chinese Academy of Sciences (CAS).

## Author Contributions

ML designed the research. XW performed the experiments. XW, ZW, HP, and JQ analyzed the data. XW, DL, and ZX collected samples. YS was responsible for the samples. XW, ZW, HP, and ML wrote the manuscript. All authors critically reviewed and approved the manuscript.

## Conflict of Interest

The authors declare that the research was conducted in the absence of any commercial or financial relationships that could be construed as a potential conflict of interest.

## Publisher’s Note

All claims expressed in this article are solely those of the authors and do not necessarily represent those of their affiliated organizations, or those of the publisher, the editors and the reviewers. Any product that may be evaluated in this article, or claim that may be made by its manufacturer, is not guaranteed or endorsed by the publisher.

## Supplementary Material

Supplementary Figure 1 Additional figures that are supplementary to this study. **(A)** Compositional bar of four cohorts. **(B)** Correlation test of four cohorts. **(C)** Dendrogram of CRr, CMm, and Hum. **(D)** Bacteria shared among CRr, CMm, and Hum.

Supplementary Table 1 Sample information **(A)** and metadata **(B)** of each cohort analyzed in the study.

Supplementary Table 2 Relative abundance of each taxonomy clade of each cohort (**A**: CRr and WRr; **B**: CMm; **C**: Hum) analyzed in the study.

Supplementary Table 3 Relative abundance of K-numbers of captive and wild *R. roxellana* in the study.

Supplementary Table 4 List of significantly differentially abundant taxon clades and K-numbers (**A**: significantly differentially abundant taxon clades; **B**: significantly differentially abundant K-numbers; **C**: significantly differentially abundant pathways) between captive and wild *R. roxellana*.
