# Supplementary figures and images for: Captivity Influences the Gut Microbiome of Rhinopithecus roxellana
Source: Front Microbiol. 2021 Dec 7;12:763022. doi: 10.3389/fmicb.2021.763022 (PMC8689068; doi:10.3389/fmicb.2021.763022)

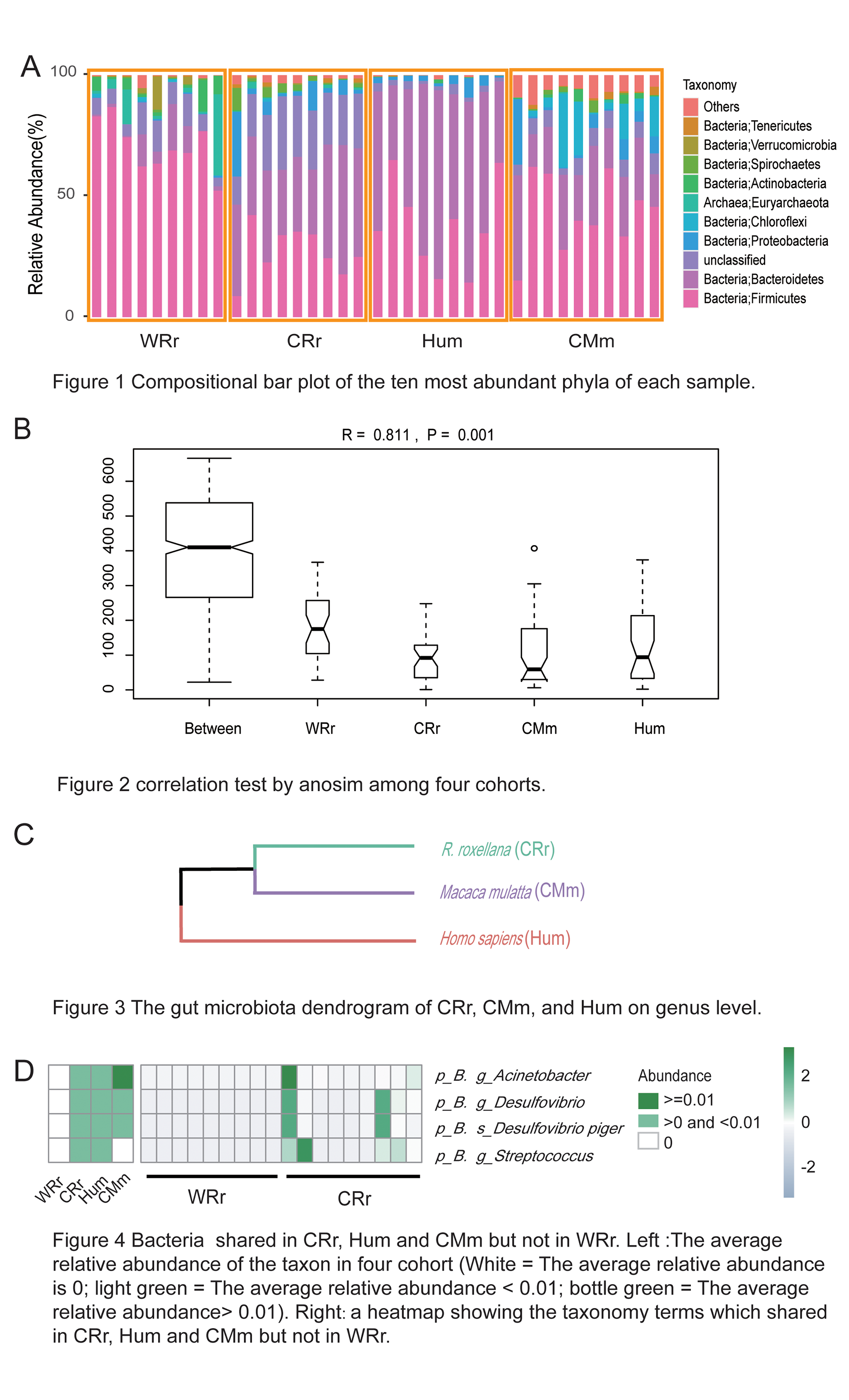

Supplement: Supplementary Figure 1 — Additional figures that are supplementary to this study. (A) Compositional bar of four cohorts. (B) Correlation test of four cohorts. (C) Dendrogram of CRr, CMm, and Hum. (D) Bacteria shared among CRr, CMm, and Hum. [file Image_1.TIF]
